# Bone health in children with long–term idiopathic subclinical hypothyroidism

**DOI:** 10.1186/1824-7288-38-56

**Published:** 2012-10-22

**Authors:** Raffaella Di Mase, Manuela Cerbone, Nicola Improda, Andrea Esposito, Donatella Capalbo, Ciro Mainolfi, Francesca Santamaria, Claudio Pignata, Mariacarolina Salerno

**Affiliations:** 1 Department of Pediatrics, University of Naples, Federico II, Naples, Italy; 2 Department of Radiology, University of Naples, Federico II, Naples, Italy

**Keywords:** Bone, Bone Mineral density, DXA, QUS, Subclinical hypothyroidism

## Abstract

**Background:**

Subclinical hypothyroidism (SH) is a relatively common condition characterized by a mild persistent thyroid failure. The management of children with SH is still a controversial issue and the decision to treat with L-thyroxine represents a clinical dilemma. Thyroid hormone and TSH play an important role in skeletal growth and bone mineral homeostasis.

**Aim:**

To evaluate whether untreated idiopathic SH may affect bone health in childhood and to compare two different diagnostic tools such as dual-energy X-ray densitometry (DXA) and quantitative ultrasound (QUS).

**Patients and Methods:**

Twenty-five children and adolescents (11 males) aged 9.8 ± 3.5 years (range 4.2-18.7) with untreated idiopathic SH were enrolled in the study. SH was diagnosed on the basis of normal FT4 levels with TSH concentrations between 4.2 and 10 mU/l. Children have been followed for 3.3 ± 0.3 years from the time of SH diagnosis. Twenty-five healthy children, age- and sex-matched, were enrolled as controls. Patients and controls underwent DXA to evaluate lumbar spine bone mineral density (BMD) and QUS at proximal phalanges of the non-dominant hand to assess bone quality, measured as amplitude-dependent speed of sound (Ad-SoS) and bone transmission time (BTT).

**Results:**

Mean BMD Z-score was −0.4 ± 1.36 in patients and −0.2 ± 1.2 in controls. Mean Ad-SoS Z-score was 0.01 ± 1.0 in patients and 0.1 ± 1.2 in controls and mean BTT Z-score was −0.03 ± 0.8 and 0.04 ± 1.1 respectively. All values were within the normal range, both in patients and in controls. There were no statistically significant differences between the two groups.

**Conclusion:**

Bone health, evaluated by lumbar spine DXA and phalangeal QUS, is not impaired in our children, despite long-term duration of idiopathic SH. Data about bone status provided by QUS are comparable to those provided by DXA. Therefore, QUS may represent a good, cheaper and safe screening test for bone evaluation in children with SH.

## Background

Subclinical hypothyroidism (SH) is defined by the serum TSH concentration above the upper limit of the reference range and serum free T4 concentration within the reference range [1]. The prevalence is comprised between 4 and 20% of the adult population [2] and about 1.7 % in US children [3]. Although in adults mild clinical signs or symptoms of hypothyroidism have been reported in some cases, there is no full consensus about the management of these subjects. Moreover, there is no clear evidence of beneficial effect of L-thyroxine treatment [4]. The management of children with SH is an even more controversial issue. Recent data indicated that idiopathic SH is a benign condition that in the majority of cases does not progress into overt hypothyroidism [5,6] and does not affect linear growth [7,8].

Thyroid hormone plays a critical role in postnatal skeletal development and in bone remodeling, influencing osteoclast-induced bone resorption and osteoblast-induced bone formation [9]. Hyperthyroidism increases bone turnover and reduces the remodeling cycle time leading to an increased risk of osteoporosis and fractures in overt and subclinical hyperthyroid patients [10-16]. As for the effect of hypothyroidism on bone, hystomorphometry data indicated that hypothyroidism significantly prolongs the bone remodeling cycle resulting in a reduced bone turnover and a gain in bone mass and mineralization with consequent significantly greater thickness in cortical bone [17]. In keeping with this data, several studies have reported on an increased BMD in adults with high or high-normal TSH, along with a positive correlation between BMD and TSH levels [18-20]. Furthermore, higher TSH levels were reported to be protective against fractures [14]. Conversely, other studies on large adult population documented an increased risk of fractures in adults with overt hypothyroidism [21,22]. Current data from in vitro studies suggest that TSH, through its receptor, has a direct effect on both components of skeletal remodeling, osteoblastic bone formation and osteoclastic bone resorption [23]. The TSH receptor (TSHR) is a member of the seven transmembrane G-protein-coupled receptor family that also includes the calcitonin and parathyroid hormone (PTH) receptors, both regulators of bone turnover [24]. TSHR knockout mice have severe osteoporosis. Heterozygous TSHR^+/−^ mice, despite normal T4 levels have significant decrease in bone density. This finding suggests that also a mild increase in TSH levels may influence bone metabolism, particularly in non-autoimmune subclinical hypothyroidism that may also be related to inactivating mutation of TSHR. Studies on bone health in untreated SH in childhood and adolescence are lacking. The most important factor in the prevention of osteoporosis is the attainment of an optimal peak bone mass, which is mainly reached during late adolescent and early adulthood [25]. Therefore, measurement of bone mineral status in childhood and adolescence may help identifying subjects, who may be exposed to an increased risk of osteoporosis later in adulthood.

Aim of this study was to evaluate whether untreated SH may affect bone health assessed by two different diagnostic tools such as dual-energy X-ray densitometry (DXA) and quantitative ultrasound (QUS) in childhood.

## Patients and methods

### Patients

The study population consisted of 25 Caucasian children (11 males), aged 9.8 ± 3.5 years (range 4.2-18.7) with untreated idiopathic SH. The diagnosis of idiopathic SH was made on the basis of the following criteria: serum TSH concentration between 4.5 mU/l and the conventional limit of 10 mU/l with a serum free thyroxine (FT4) concentration within the reference range, along with the absence of anti-thyroglobulin (Tg-Ab) and anti-thyroperoxidase antibodies (TPO-Ab), normal echogenicity of the parenchyma on thyroid ultrasound and adequate urinary iodine excretion. The criteria for enrollment also included the persistence of a similar biochemical pattern of SH for at least 2 years prior to entering into the study and the absence of palpable goiter or symptoms related to hypothyroidism.

In 13**/**25 SH children, thyroid function measurement had been made for short stature (5 cases), obesity (1 case) or familial history of thyroid diseases (7 cases). In the remaining 12 cases, TSH measurement had been performed as part of a check-up. From the first finding of SH until the time of this study, all children were periodically followed at our center. At enrollment in the study, the duration of SH was 4.6 ± 2.8 (range 2.0-13.3) years. Six/25 subjects were pubertal. None of them was ever been treated with L-thyroxine. Twenty-five healthy euthyroid children (14 males), matched for age, sex, and pubertal status, who had undergone a check-up including also TSH measurement participated in the study as controls.

### Study protocol

At the entry into the study, all subjects underwent to clinical examination, anthropometric measurements, determination of urinary iodine, fasting blood sample for calcium (Ca), phosphorus (P), alkaline phosphates (ALP), parathyroid hormone (PTH), TSH, FT4, thyroglobuline (Tg), Tg-Ab, TPO-Ab, insulin-like growth factor 1 (IGF1), bone age (BA) assessment and bone status evaluation. Bone maturation was evaluated using the Greulich and Pyle method and was expressed as BA/chronological age (CA) ratio. Moreover, all participants answered questionnaires on lifestyle and health related topics including questions on physical activity, smoking habits, intake of calcium, former or current diseases and use of medications.

The study protocol was approved by the Local Ethical Committee and written informed consent to participate into the study was obtained from the all subjects’ parents when the chronological age was lower than 18 and directly from each older subject.

### Anthropometric data

Standing height was measured using a mechanical stadiometer to the nearest 0.1 cm and body weight was measured with a mechanical balance to the nearest 0.1 kg. The body mass index (BMI) was calculated as weight/height^2^. Parental height was measured and target height (TH) was calculated according to Tanner et al. [26]. Height, TH and BMI were also expressed as Z-score, adjusted for age and gender in accordance with Italian standards [27].

### Laboratory data

A venous blood sample was drawn from all participants at 8.00 in the morning after a 12-hour fast. TSH, FT4, Tg, Tg-Ab, TPO-Ab and PTH serum concentrations were measured by electrochemiluminescence immunoassay (ECLIA) using a commercial kit (Elecsys ecobas e. Roche Diagnostics) (reference ranges: TSH, 0.3–4.2 mU/l; FT4, 0.9–1.7 ng/dl; Tg, 0–50 ng/ml; Tg-Ab, 0–115 mIU/l; TPO-Ab, 0–34 mIU/l; PTH, 10–75 pg/ml). Serum IGF1 levels were measured using a two-site IRMA kit (Diagnostics System Laboratories, Inc., Webster, TX, USA). The IGF1 intra- and inter-assay coefficients of variation (CV) were 3.4 and 8.2% respectively. The values obtained were standardized by age and sex and expressed in SDS. Urine iodine levels were analyzed with an automated system (Autoanalyzer 3 system, Bran CLuebbe GmbH, Nordestedt, Germany) (reference range: 100–200 mg/l). The other parameters were measured by a standardized automatic colorimetric method using the Cobas Integra 400 Analyzer (USA).

### Imaging studies

In all subjects bone status was evaluated by DXA, the most widespread diagnostic tool to assess bone health, and by QUS. Bone mineral density was measured by DXA at the lumbar spine from the first to the fourth lumbar vertebra (L1–L4), using a Hologic QDR 1000 densitometer (Hologic, Waltham, MA, USA). Daily calibrations of the densitometer performed with a phantom during a 1-year period had provided a coefficient of variation of 0.56%. All examinations were carried out in the continuous presence of trained technicians. BMD results were expressed as g/cm^2^ and as BMD Z-score calculated on the basis of the normal reference values for age and sex provided by the DXA system manufacturer. Bone quality was assessed in each subject by QUS measurements performed with a DBM Sonic 1200 bone profiler (Igea S.r.l., Carpi, MO, Italy) employing a sound frequency of 1.25 MHz. QUS is an easy, cheap and radiation-free technique to evaluate bone mineral status at peripheral skeleton through amplitude-dependent speed of sound (Ad-SoS), that expresses the ultrasound velocity inside the bone, and bone transmission time (BTT), reflecting the bone characteristics without the interference of the soft tissue. QUS was performed on the second to the fifth proximal phalanges of the non-dominant hand and the mean value per person was calculated. Measurements were performed by the same operator and the coefficient of variation was 0.73%, determined by repeated measurements in a subgroup of 12 subjects (three measurements per person on three different days). Ad-SoS and BTT results were expressed as Z-scores calculated on the basis of the normal values for age and sex obtained in a large Italian population sample [28].

In agreement with the International Society for Clinical Densitometry [29], BMD was considered to be normal when values were above −2 Z-score , a similar cut-off was used for Ad-SoS and BTT.

### Statistical analysis

The statistical analyses were conducted using SPSS for Windows, version 15.0 (SPSS Inc., Chicago, IL, USA). All data are expressed as mean ± S.D. Comparison between patients and controls for all variables was performed by paired Student’s *t-test* or Wilcoxon matched pairs test as appropriate. Pearson’s correlation coefficient was used to evaluate the relationship between variables. Multiple stepwise regression analysis was used to evaluate the effect of age, sex, BMI, thyroid hormone status and duration of SH on BMD, Ad-SoS and BTT. Differences were considered statistically significant when P <0.05.

## Results

**Figure 1:**
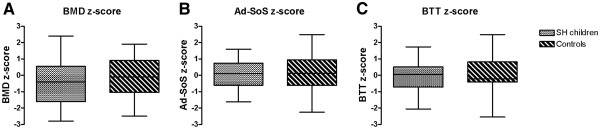
**Bone mineral density (BMD) (A), and bone quality expressed by Ad-SoS (B) and BTT (C) Z-scores in children with subclinical hypothyroidism compared with the control group.** (The boxes show medians, 25^th^ and 75^th^ percentiles, and the whiskers represent the highest and lowest values).

Both SH and control children reported mild to moderate physical activity. None of them were smokers. Daily calcium intake resulted below the recommended intake of 1300 mg/day [30] in the majority of subjects in both groups (820±272 mg/day vs 862±352 mg/day in SH and controls, respectively, p=n.s.). None had any other disease or was taking any medications known to affect bone metabolism. Clinical, biochemical and hormonal data as well as BMD and QUS results of children with SH compared with controls are reported in Table 1. Mean lumbar BMD Z-score in SH children (−0.4 ± 1.36) and in controls (−0.2 ± 1.2) was within the normal range, without any significant difference within the two groups (Figure 1). Two**/**25 SH children (8%) showed a BMD Z-score < −2, however no significant difference between these 2 children and the remaining group of SH children was detected. Moreover, the same percentage of children with reduced BMD was also detected in the controls.

**Table 1:** Main clinical, biochemical and bone health data in children with SH and in controls

	**SH children**	**Controls**	**P**
Age (years)	9,8 ± 3,5	10,9 ± 2,7	ns
H Z-score	−0,37 ± 1,33	−0,60 ± 1,49	ns
TH Z-score	−0,83 ± 0,92	−0,79 ± 0,87	ns
BMI Z-score	0,32 ± 1,23	−0,52 ± 1,2	< 0,05
EO/EC	0,93 ± 0,13	na	na
TSH (IU/l)	6,39 ± 1,25	2,84 ± 0,92	< 0,0001
FT4 (ng/dl)	1,28 ± 0,13	1,27 ± 0,10	ns
IGF-1 Z-score	−0,12 ± 1,39	−0,30 ± 0,91	ns
Ca (mg/dl)	9,80 ± 0,37	9,79 ± 0,29	ns
P (mg/dl)	4,90 ± 0,51	4,80 ± 0,38	ns
ALP (IU/l)	203,20 ± 75,95	209,76 ± 65,48	ns
PTH (pg/ml)	36,77 ± 12,65	41,52 ± 25,28	ns
BMD Z-score	−0,4 ± 1,36	−0,2 ± 1,2	ns
Ad-SoS Z-score	0,01 ± 1	0,1 ± 1,2	ns
BTT Z-score	−0,03 ± 0,8	0,04 ± 1,1	ns

As for the results of QUS, mean Ad-SoS Z-score as well as mean BTT Z-score were normal in SH children (0.01 ± 1.0 and −0.03 ± 0.8, respectively) and not significantly different from the controls (0.1 ± 1.2 and 0.04 ± 1.1, respectively) (Figure 1)**.** All these parameters still were comparable even when they were corrected for chronological age. No difference was found in BMD, Ad-SoS and BTT Z-scores between males and females and between prepubertal and pubertal subjects in SH children (data not shown). Biochemical parameters of bone turnover, Ca, P, ALP and PTH levels, were normal and comparable in both groups. As expected, in SH children TSH levels were significantly higher than in controls, ranging from 4.58 IU/L to 8.88 IU/L, free T4 concentration were similar and within the normal range in both groups. BMI was significantly higher in SH children than in controls (p = 0.02) and 2/25 SH children were obese (BMI > 2 SDS). However, no relationship was detected between BMI-SDS and BMD Z-score, Ad-SoS and BTT Z-scores. A positive relationship was detected between height and BMD Z-score as expected (r = 0.59, p <0.02). Moreover, BMD Z-score was positively correlated with both Ad-SoS and BTT Z-scores (r = 0.42, p <0.04 and r = 0.52, p <0.008, respectively).

After multiple regression analysis, neither TSH levels at study entry or the duration of SH significantly affected BMD, Ad-SoS and BTT Z-scores.

## Discussion

In this study we evaluated whether subclinical hypothyroidism in children may impact on biochemical markers of bone metabolism, and on bone structure and quality. Compared to children with TSH within normal range, biochemical markers of bone metabolism and bone health, evaluated by both bone mineral density and bone quality, were not impaired in children with untreated long lasting idiopathic SH.

The peak bone mass is mainly reached during adolescence or early adulthood and, therefore, any factor affecting bone growth and remodeling or bone storage during childhood might impair the attainment of an optimal peak bone mass, predisposing to osteoporosis and fracture later in life.

It is well recognized that along with genetic, ethnic, nutritional and hormonal factors as well as lifestyle and physical activities, also thyroid hormone and TSH play an important role in skeletal growth and bone mineral homeostasis. Children with untreated hypothyroidism may have delayed skeletal maturation and reduced growth velocity leading to short stature, whereas no data on bone health are available. However, no impairment in bone health evaluated by DXA and QUS was observed in congenital hypothyroid children and adolescents after long-term L-thyroxine replacement therapy [31,32]. Conversely, in another study BMD was normal in treated children with congenital hypothyroidism but slightly lower than healthy controls [33]. In our SH children height was normal, within the target height, and skeletal maturation was adequate to the chronological age. None of them showed clinical signs or symptoms of hypothyroidism such as goiter, fatigue, mood changes or impaired concentration. Data on the effect of subclinical hypothyroidism on bone mineral status in children are completely lacking, while a few studies in adults yielded conflicting results. In the Hunt study [34], not statistically significant differences in BMD were found if TSH was within or above the reference range. Conversely, Grimmes et al. in the Tromso study showed that postmenopausal women with serum TSH above the 97.5 percentile had a BMD significantly higher than women with serum TSH within the normal range [20]. This data is in agreement with the results reported by Bertoli et al. [18] of a mean leg BMD higher than controls in subclinical premenopausal women. However, other studies have shown a reduced BMD and an increased risk of fracture in adults with SH [35,36]. In a recent study Lee et al. [36] evaluated the risk of hip fractures in a prospective cohort study of 3567 adults aged 65 years or older with subclinical thyroid dysfunction. The results of the study showed that men with high TSH levels, but lower than 10 mIU/L, had an increased risk of fracture, thus suggesting that even mild subclinical hypothyroidism may have deleterious effects on bone. Moreover, Nagata et al. [37] recently reported that calcaneus osteosonographic assessment indices of right feet, measured using the quantitative ultrasound, were lower in postmenopausal women with SH than in controls.

To our knowledge, this is the first study exploring the bone mineral status in children with SH. Our results indicate that SH children have a normal bone density and structure as assessed by lumbar DXA and phalangeal QUS. Both Ad-SoS and BTT were normal and comparable to the controls, despite the long lasting SH. Moreover, our study indicates that phalangeal QUS is a reliable tool to evaluate bone health, since a good correlation was found with DXA, suggesting that QUS may represent an easy and useful tool for the bone health screening in children with SH.

## Conclusion

In this study we showed that bone health assessed at lumbar spine by DXA and at phalanges of the hand by QUS, was not affected in long term untreated SH children and adolescents.

## Abbreviations

SH: Subclinical hypothyroidism; QUS: Quantitative ultrasound; BMD: Bone mineral density; BTT: Bone transmission time; Ad-SoS: Amplitude-dependent speed of sound; TSHR: TSH receptor; Tg-Ab: Anti-thyroglobulin antibodies; TPO-Ab: Anti-thyroperoxidase antibodies; BA: Bone age; CA: Chronological age; SDS: Standard deviation score; DXA: Dual-energy X-ray densitometry.

## Competing interests

The authors declare that they have no competing interests.

## Authors’ contribution

All authors have equally participated in drafting of the manuscript and/or critical revision of the manuscript for important intellectual content. All authors read and approved the final manuscript.
